# Sequence variants selected from a multi-breed GWAS can improve the reliability of genomic predictions in dairy cattle

**DOI:** 10.1186/s12711-016-0259-0

**Published:** 2016-11-04

**Authors:** Irene van den Berg, Didier Boichard, Mogens S. Lund

**Affiliations:** 1 Department of Molecular Biology and Genetics, Faculty of Science and Technology, Center for Quantitative Genetics and Genomics, Aarhus University, 8830 Tjele, Denmark; 2 GABI, INRA, AgroParisTech, Université Paris Saclay, 78350 Jouy-en-Josas, France

## Abstract

**Background:**

Sequence data can potentially increase the reliability of genomic predictions, because such data include causative mutations instead of relying on linkage disequilibrium (LD) between causative mutations and prediction variants. However, the location of the causative mutations is not known, and the presence of many variants that are in low LD with the causative mutations may reduce prediction reliability. Our objective was to investigate whether the use of variants at quantitative trait loci (QTL) that are identified in a multi-breed genome-wide association study (GWAS) for milk, fat and protein yield would increase the reliability of within- and multi-breed genomic predictions in Holstein, Jersey and Danish Red cattle. A wide range of scenarios that test different strategies to select prediction markers, for both within-breed and multi-breed prediction, were compared.

**Results:**

For all breeds and traits, the use of variants selected from a multi-breed GWAS resulted in substantial increases in prediction reliabilities compared to within-breed prediction using a 50 K SNP array. Reliabilities depended highly on the choice of the prediction markers, and the scenario that led to the highest reliability varied between breeds and traits. While genomic correlations across breeds were low for genome-wide sequence variants, the effects of the QTL variants that yielded the highest reliabilities were highly correlated across breeds.

**Conclusions:**

Our results show that the use of sequence variants, which are located near peaks of QTL that are detected in a multi-breed GWAS, can increase reliability of genomic predictions.

**Electronic supplementary material:**

The online version of this article (doi:10.1186/s12711-016-0259-0) contains supplementary material, which is available to authorized users.

## Background

Accuracy of genomic predictions is highly influenced by the size of the reference population used [1–3]. In cattle, for breeds such as the Holstein breed, this is not a problem since large reference populations are available at both the national and international levels [4], but for breeds with a smaller reference populations, accuracies of genomic prediction may not be sufficiently high. Using a large multi-breed reference population could potentially increase the accuracy of genomic predictions, by allowing breeds that have a small reference population to use information from other breeds. However, in practice, large increases in accuracy of genomic predictions are obtained only when the breeds included in the multi-breed reference population are closely related [5, 6]. When more distant breeds are combined together, increases in accuracies of genomic predictions are generally small or zero compared to within-breed predictions [7–11]. One reason for this could be that linkage disequilibrium (LD) is conserved over much shorter distances across breeds than within breeds [12]. With the availability of high-density single nucleotide polymorphism (SNP) chips, de Roos et al. [12] showed that the LD between single nucleotide polymorphisms (SNPs) on the high-density SNP chip across dairy cattle breeds is sufficiently high to make across-breed prediction feasible and it was then assumed that increasing marker density furthermore to the whole-genome sequence level would improve multi-breed prediction. However, reliabilities of genomic predictions that are obtained with the bovine high-density SNP chip (HD) are not much higher than those with the 50 K SNP chip [9, 10]. Increasing marker density to the HD or the sequence level adds a large number of genome-wide variants but only a few of these variants are close to the causative mutations. Unless only variants in perfect LD with the causative mutations are used, the variants in imperfect LD with the causative mutations will limit the reliability of genomic predictions [13]. While whole-genome sequence data contain causative mutations and variants in high LD with some causative mutations, most of the variants are in low LD with the causative mutations. Thus, it is not surprising, that the use of whole-genome sequence data for genomic prediction does not necessarily increase reliabilities of genomic predictions compared to the use of genome-wide SNPs [14, 15], especially if the models used do not allow for sufficiently different within-breed variances and across-breed covariances for different SNPs.

In a simulation study, Pérez-Enciso et al. [16] obtained very high reliabilities by including the causative mutations in the model, while either addition of non-causative variants or removal of some causative mutations decreased reliabilities. Studies in cattle [17, 18] and *Drosophila melanogaster* [19] showed that selecting prediction variants based on the results of genome-wide association studies (GWAS) can yield substantial increases in the reliability of genomic predictions.

Because LD is conserved over much shorter distances across than within breeds [12], increasing the distance between causative mutations and prediction variants had a stronger effect on across-breed prediction than on within-breed prediction. In a simulation study [20], reliability of genomic predictions decreased faster across breeds than within breeds as the distance between prediction variants and causative mutations increased. Therefore, in order to infer information across breeds, it is important to use variants that are in high LD with the causative mutations. Although the true causative mutations are unknown, with a few exceptions [21], a large number of quantitative trait loci (QTL) regions have been detected in dairy cattle [22–27], and this information could be used to select sequence variants for genomic prediction. However, variants that are linked to a QTL in one breed but not in another breed can introduce noise, and reduce accuracy of genomic prediction for the other breed. Thus, careful selection of QTL variants is likely to be relevant for multi-breed prediction. Because LD is conserved over shorter distances across breeds, fewer variants are associated with the same causative mutations across breeds. Consequently, multi-breed GWAS results in more precise QTL mapping for variants that are shared across breeds [11, 28, 29].

Another potential difficulty in multi-breed prediction is that variant effects differ across breeds, which can be due to dominance or epistasis. However, even for genes with additive effects, differences in effects could be due to allele frequencies differing among breeds, or simply to the LD between prediction variants and causative mutations differing among breeds [6]. Thus, considering that SNP effects can be correlated across breeds rather than assuming that they are the same in each breed may be important to take advantage of sequence data for genomic prediction.

When within-breed genomic predictions are used, they rely heavily on the structure of the relationships within the breed that create LD in relatively large regions. Such structures are disrupted when populations from different breeds are combined, which results in LD being persistent over shorter regions across breeds. In addition, SNP effects can be easily dominated by the SNP effects in the breed with the largest population, which may lead to the prediction of a non-existing effect in the other breeds. As a consequence, the SNP may lose its predictive ability for the other breeds or even introduce noise from the breed with the largest reference population. Thus, in order to allow for private genetic variation and efficient use of within-breed family relationships, it could be useful to include a genomic component that models the genomic covariances within a given breed in the model.

Our objective was to investigate whether the use of variants at QTL that are selected from a multi-breed GWAS for milk, fat and protein yields would increase the reliability of within- and multi-breed genomic predictions in three dairy cattle breeds that range from very related populations to unrelated breeds. We used a model with a 50 K SNP genomic component and a QTL genomic component that includes sequence variants. We assumed that reliability of genomic predictions would increase when QTL variants were included in the model compared to models using only 50 K SNPs and that if too many were included, this advantage would decrease. More precisely, we expected that:

1. single-trait models that assume equal variant effects across breeds would be efficient for closely related populations;
2. including a QTL component with sequence variants would increase the reliability of genomic predictions and increase the correlations of variant effects between breeds compared to the 50 K SNP component;
3. a restricted number of prediction markers per QTL interval would improve the reliability of genomic predictions, especially for distantly related breeds;
4. a multi-breed GWAS would select sequence variants more accurately than a within-breed GWAS, especially for multi-breed prediction.

We used different models to test these assumptions.

## Methods

### Data

All genotype and phenotype data used in this study were obtained from pre-existing routine genetic evaluation data for the dairy cattle populations and required no ethical approval. Data from 5852 French Holstein (HOLFR), 5411 Danish Holstein (HOLDK), 1203 Danish Jersey (JER) and 937 Danish Red (RDC) bulls were included in the analyses. Although the HOLFR and HOLDK populations belong to the same breed, they were considered as different breeds. Holstein and RDC breeds are weakly related, while the JER breed is much more distantly related from either the RDC or Holstein breeds [6]. For all the bulls, deregressed proofs (DRP) were available for milk, fat and protein yields. Since the French and Danish scales differ, it was necessary to standardize the DRP within each breed, so that they were comparable between countries. All individuals were genotyped with the 50 K SNP chip and a subset of the individuals was also genotyped with the HD SNP chip, or sequenced. Individuals that were genotyped with the 50 K SNP chip were first imputed to HD, and then to the whole-genome sequence level, so that full genome sequence information was available for all the individuals. Imputation of Danish bulls from 50 K to HD and imputation of both French and Danish bulls from HD to whole-genome sequence level were done by using IMPUTE2 [30], while imputation of French bulls from 50 K to HD was performed by using Beagle [31]. For the Danish bulls, imputation from HD to whole-genome sequence level was based on a multi-breed reference population that included 1228 individuals from the fourth run of the 1000 Bull Genomes project [32] and 80 bulls from other projects carried out at Aarhus University. The HOLFR bulls were imputed by using a joint multi-breed French-Danish reference population that included 122 Holstein, 27 Jersey, 28 Montbéliarde, 23 Normande and 45 Danish Red bulls. More details on the imputation of the Danish bulls are in Brøndum et al. [17] and for the imputation of the French bulls from 50 K to HD in Hozé et al. [33].

For each population, individuals were divided into a training and a validation population. The validation populations consisted of the youngest individuals of each breed, and their sires were excluded from the training population. The training populations included 4911 HOLDK, 5335 HOLFR, 957 JER and 745 RDC bulls, and the validation populations consisted of 500 HOLDK, 517 HOLFR, 246 JER and 192 RDC bulls.

### Selection of prediction markers included in the QTL component

Several scenarios with different sets of prediction markers and different models were investigated. All sets of prediction markers included only variants with a minor allele frequency (MAF) higher than 0.01 and an IMPUTE2 INFO score of at least 0.9, which resulted in the basic set (50 K) comprising 37,856 SNPs from the 50 K SNP chip. For the other sets, variants were selected based on their associations with milk, fat or protein yield that had been identified in previously performed GWAS.

The dataset used for the multi-breed GWAS included all the bulls of the four populations (HOLFR, HOLDK, JER and RDC) in the training populations, their sires, and an additional 1935 Montbéliarde and 1725 Normande bulls. First, a GWAS was performed within each of the six populations, using whole-genome sequence data. After filtering out variants with a MAF lower than 0.005 and an IMPUTE2 INFO score less than 0.60, 24,550,115 SNPs and indels remained in the dataset. A single-marker model was run for each of these polymorphisms, within each of the six populations:

$$y_{ik} = \mu + s_{ik} + \beta g_{i} + e_{ik} ,$$

where $y_{ik}$ is the DRP of milk yield, fat yield or protein yield for individual $i$ with sire $k$, $s_{ik}$ the random effect of sire $k$, $\beta$ the effect of the variant, $g_{i}$ the allele dose (ranging from 0 to 2) for individual $i$ and $e_{ik}$ a random residual.

Subsequently, a multi-breed GWAS was performed combining all six populations. To reduce computing time, the multi-breed GWAS was only run for variants with a *p* value <10^−5^ for the HOLDK or HOLFR bulls, or <10^−3^ for one of the other breeds for at least one of the traits. A breed effect was added to the model to account for between-breed differences:

$$y_{ijk} = \mu + s_{ik} + b_{ij} + \beta g_{ijk} + e_{ijk} ,$$

where $b_{ij}$ is the effect of breed $j$ of individual $i$. A full description of the GWAS is in [29].

Within breeds, variants were selected based on their associations with milk, fat or protein yield, which had been identified in either the within-breed or multi-breed GWAS, while for multi-breed analyses, variants were selected based on their associations with milk, fat or protein yield, which were detected in the multi-breed GWAS. Thresholds for within-breed p values were equal to 10^−*t*^, with *t* equal to 10, 12 or 14 for Holstein populations and 4, 6 or 8 for Jersey and Danish Red populations. For the multi-breed models, *t* was equal to 10, 14 or 20. Due to the large differences in number of individuals per breed, the power of the GWAS varied strongly between breeds. Therefore, different thresholds were used for each breed, i.e. the thresholds for the JER and RDC breeds were chosen so that the range of the number of selected variants included the number of variants used for the HOLDK and HOLFR populations. An overview of all scenarios can be found in Table 1. Within breeds (WB-50 K + QTLt scenario), all variants that passed these thresholds were selected. Subsequently, LD pruning was performed on the selected variants using PLINK [34], with a R^2^ threshold of 0.95. Selection of variants was the same for the multi-breed and within-breed analyses in the MB-50 K + QTLt scenarios. In scenarios MB-50 K + QTLt-n/w, the number of variants per interval ($n$) was, after LD pruning, limited to the 1, 10 or 25 variants with the lowest p values, per window ($w$) of 1, 2 or 10 Mb. Intervals were defined starting from the highest peak, until there were no more variants with a p value below *t*. The number of QTL variants selected from the within- and multi-breed GWAS are in Tables 2 and 3, respectively. If a variant was included in the QTL component of one scenario, it was excluded from the 50 K component for that scenario.

**Table 1:** Descriptions of the scenarios used in the paper

Scenario^a^	Model	QTL component^b^
WB-50 K	WB	–
WB-50 K + WBQTLt	WB	All variants with a p value below 10^−*t*^ in a within breed GWAS
WB-50 K + MBQTLt	WB	All variants with a p value below 10^−*t*^ in a multi breed GWAS
WB-50 K + MBQTLt-n/w	WB	Maximum *n* variants with a p value below 10^−*t*^ per interval of *i* Mb in a multi breed GWAS
MB-50 K	MB	–
MB-50 K + MBQTLt	MB	All variants with a p value below 10^−*t*^ in a multi breed GWAS
MB-50 K + MBQTLt-n/w	MB	Maximum *n* variants with a p value below 10^−*t*^ per interval of *i* Mb in a multi breed GWAS
MT-50 K	MT	–
MT-50 K + MBQTLt	MT	All variants with a p value below 10^−*t*^ in a multi breed GWAS
MT-50 K + MBQTLt-n/w	MT	Maximum *n* variants with a p value below 10^−*t*^ per interval of *i* Mb in a multi breed GWAS

*WB* within-breed, *MB* multi-breed,*MT* multi-trait model

^a^Acronym of the scenario

^b^Describes how the variants in the QTL component were selected

**Table 2:** Different sets of QTL markers selected from within-breed GWAS

Set	Selection threshold	Number of selected variants		
		Milk yield	Fat yield	Protein yield
*Danish Holstein*				
WBQTL10	10^−10^	2595	2523	1491
WBQTL12	10^−12^	1868	1719	612
WBQTL14	10^−14^	1511	1220	298
*French Holstein*				
WBQTL10	10^−10^	2249	1924	921
WBQTL12	10^−12^	1382	1108	330
WBQTL14	10^−14^	958	782	168
*Jersey*				
WBQTL4	10^−04^	14,101	6632	3219
WBQTL6	10^−06^	2464	578	345
WBQTL8	10^−08^	677	51	22
*Danish Red*				
WBQTL4	10^−04^	9548	4925	5330
WBQTL6	10^−06^	873	648	383
WBQTL8	10^−08^	80	232	12

**Table 3:** Different sets of QTL markers selected from multi-breed GWAS

Set	Selection threshold	Window size (Mb)	n^a^	Number of selected variants		
				Milk yield	Fat yield	Protein yield
MBQTL10	10	–	–	8361	9615	6119
MBQTL10-1/1	10	1	1	375	448	522
MBQTL10-10/1	10	1	10	1954	2612	2773
MBQTL10-25/1	10	1	25	3130	4190	4096
MBQTL10-1/2	10	2	1	269	292	342
MBQTL10-10/2	10	2	10	1457	1856	2080
MBQTL10-25/2	10	2	25	2363	3189	3410
MBQTL10-1/10	10	10	1	111	109	107
MBQTL10-10/10	10	10	10	709	775	911
MBQTL10-25/10	10	10	25	1230	1454	1808
MBQTL14	14	–	–	3821	4077	1402
MBQTL14-1/1	14	1	1	102	155	134
MBQTL14-10/1	14	1	10	614	816	633
MBQTL14-25/1	14	1	25	1046	1341	894
MBQTL14-1/2	14	2	1	67	111	95
MBQTL14-10/2	14	2	10	416	635	534
MBQTL14-25/2	14	2	25	762	1065	801
MBQTL14-1/10	14	10	1	27	40	41
MBQTL14-10/10	14	10	10	194	279	295
MBQTL14-25/10	14	10	25	352	534	517
MBQTL20	20	–	–	2225	2252	299
MBQTL20-1/1	20	1	1	30	45	23
MBQTL20-10/1	20	1	10	203	251	130
MBQTL20-25/1	20	1	25	384	424	205
MBQTL20-1/2	20	2	1	18	35	19
MBQTL20-10/2	20	2	10	138	192	104
MBQTL20-25/2	20	2	25	257	314	162
MBQTL20-1/10	20	10	1	7	15	12
MBQTL20-10/10	20	10	10	48	94	57
MBQTL20-25/10	20	10	25	115	173	85

^a^Maximum number of variants per interval (n), and the number of selected variants for milk, fat and protein yields

### Statistical models

Genomic estimated breeding values (GEBV) were estimated using a Bayesian SNP best linear unbiased prediction (BLUP) model as implemented in the Bayz software [35], using only the 50 K data or the 50 K data and a second marker component with QTL marker components. In the models using only the 50 K data, all SNP effects were assumed to come from a single normal distribution. In the models that included a QTL component, QTL marker effects were assumed to come from a second normal distribution. Both within- and multi-breed models were tested and in the multi-breed models, the same trait in different breeds was considered either as a single trait, using a fixed breed effect to account for differences between breeds, or as multiple correlated traits, using a multi-trait model. For all scenarios, the Markov chain Monte Carlo (MCMC) was run for 50,000 iterations, discarding the first 10,000 as burn-in.

### Within-breed model with a 50 K component

In the basic model (WB-50 K), only the 50 K SNPs were used for within-breed prediction:

$$y_{i} = \mu + \mathop \sum \limits_{m = 1}^{M} z_{im} a_{m} + e_{i} ,$$

where $y_{i}$ is the deregressed proof (DRP) of individual $i$, $\mu$ the mean, $M$ is the total number of 50 K SNPs, $z_{im}$ the genotype of individual $i$ for SNP $m$, $a_{m}$ the allele substitution effect of SNP $m$ and $e_{i}$ a random residual for individual $i$. SNP effects and residuals were assumed to be drawn from normal distributions $\sim N\left( {0,\sigma_{a}^{2} } \right)$ and $\sim N\left( {0,\sigma_{e}^{2} } \right)$, respectively. Additive SNP variance $\sigma_{a}^{2}$ and residual variance $\sigma_{e}^{2}$ were assigned uniform non-informative priors.

### Within-breed models with 50 K and QTL genomic components

In scenarios WB-50 K + WBQTL and WB-50 K + MBQTL, a second genetic component was added to the model, using WBQTLt, MBQTLt or MBQTLt-n/w variants:

$$y_{i} = \mu + \mathop \sum \limits_{m = 1}^{M} z_{im} a_{m} + \mathop \sum \limits_{n}^{N} z_{in} q_{n} + e_{i} ,$$

where $N$ is the total number of QTL markers, $z_{in}$ the genotype of individual $i$ for marker $n$, and $q_{n}$ the allele substitution effect of marker $n$. QTL marker effects were drawn from a normal distribution $\sim N\left( {0,\sigma_{q}^{2} } \right)$, and additive QTL variance $\sigma_{q}^{2}$ was assigned a uniform non-informative prior.

### Multi-breed models

MB-50 K was a single-trait multi-breed model that assumed that the same trait measured in different breeds was a single trait, with a breed effect to account for the difference in means between breeds:

$$y_{i} = \mu + b_{ij} + \mathop \sum \limits_{m = 1}^{M} z_{im} a_{m} + e_{i} ,$$

where $b_{ij}$ is the effect of breed $j$ of individual $i$. A uniform non-informative prior was assigned to $b_{i}$.

Model MB-50 K + MBQTL was similar to the MB-50 K model, with the addition of one of the MBQTLt or MBQTLt-n/w sets as a multi-breed QTL component:

$$y_{i} = \mu + b_{ij} + \mathop \sum \limits_{m = 1}^{M} z_{im} a_{m} + \mathop \sum \limits_{n}^{N} z_{in} q_{n} + e_{i} .$$

### Multi-trait models

In the basic multi-trait model (MT-50 K), the same trait measured in different breeds was considered as multiple traits by assuming a correlation between allele substitution effects in the 50 K component across breeds:

$$y_{ij} = \mu_{j} + \mathop \sum \limits_{m = 1}^{M} z_{im} a_{jm} + e_{ij} ,$$

where $y_{ij}$ is the DRP of individual $i$ from breed $j$, $\mu_{j}$ the mean of breed $j$, and $a_{jm}$ the allele substitution effect of marker $m$ in breed $j$. Additive marker effects were assumed to be normally distributed $\sim N\left( {0,\sigma_{aj}^{2} } \right)$ with additive marker variance $\sigma_{aj}^{2}$. Uniform non-informative priors were assigned to $\sigma_{aj}^{2}$ and to covariance $\sigma_{aj,ak}$ between the additive marker effects on the DRP in breed $j$ and on the DRP in breed $k$. Residual covariances between DRP for individuals of different traits were 0.

Model MT-50 K + MBQTL was similar to the MT-50 K model, except for the addition of one of the MBQTLt or MBQTLt-n/w sets as a multi-breed QTL component:

$$y_{ij} = \mu_{j} + \mathop \sum \limits_{m = 1}^{M} z_{im} a_{jm} + \mathop \sum \limits_{n}^{N} z_{in} q_{jn} + e_{ij} ,$$

where $q_{jn}$ is the additive QTL marker effect of marker $n$ in breed $j$. Additive marker effects are assumed to be normally distributed $\sim N\left( {0,\sigma_{qj}^{2} } \right)$. Both $\sigma_{qj}^{2}$ and covariance $\sigma_{qj,qk}$ between the additive QTL marker effects on the DRP in breed $j$ and on the DRP in breed $k$ were assigned uniform, non-informative priors.

Genomic correlations between 50 K SNP effects on the DRP in different breeds were estimated with the MT-50 K and MT-50 K + MBQTL models, and genomic correlations between QTL marker effects on the DRP in different breeds were estimated with the MT-50 K + MBQTL model. Genomic correlations were considered significant if they were greater than twice the standard error.

### Evaluation of scenarios

Reliabilities were estimated as the squared correlation between DRP and GEBV, divided by the mean reliability of DRP in the test population. Bias was assessed by regression of DRP on GEBV. In the WB-50 K + MBQTL14-10/2 scenario for milk yield, five MCMC chains were run to assess convergence. Correlations between GEBV obtained by different runs were above 0.9999 for all breeds.

In the scenarios with a QTL component, the proportion of variants explained by the QTL component was estimated as:

$$h_{QTL}^{2} = \frac{{\sigma_{QTL}^{2} }}{{\sigma_{50K}^{2} + \sigma_{QTL}^{2} + \sigma_{e}^{2} }},$$

where $\sigma_{50K}^{2}$ and $\sigma_{QTL}^{2}$ are the variances of the 50 K and QTL components, respectively. These variances were estimated using the Gbayz programme that is part of the Bayz software [35]. For each MCMC iteration, ${\text{var}}\left( {{\mathbf{Za}}} \right)$ and ${\text{var}}\left( {{\mathbf{Zq}}} \right)$ were estimated, where ${\mathbf{Z}}$ is a design matrix and ${\mathbf{a}}$ and ${\mathbf{q}}$ are vectors of the regression coefficients of 50 K and QTL marker effects, respectively. Subsequently, posterior estimates of $\sigma_{50K}^{2}$ and $\sigma_{QTL}^{2}$ were obtained by averaging ${\text{var}}\left( {{\mathbf{Za}}} \right)$ and ${\text{var}}\left( {{\mathbf{Zq}}} \right)$ over all MCMC cycles.

## Results

### Comparison between different scenarios and prediction models

Reliabilities of genomic predictions obtained by using the 50 K SNPs and the scenarios that led to the highest reliabilities for each breed and trait are in Table 4. The highest reliabilities of genomic predictions for the HOLDK and HOLFR populations and the JER and RDC populations were obtained in scenarios MB-50 K-MBQTLt-n/w and WB-50 K-MBQTLt-n/w, respectively. Averaged across traits, the increase in reliability of the best scenario compared to model WB-50 K was equal to 0.08, 0.08, 0.06 and 0.06 for HOLDK, HOLFR, JER and RDC, respectively. The set of QTL markers that resulted in the highest reliability and the number of QTL variants in that set varied between breeds and traits. Averaged across traits, the numbers of QTL markers that yielded the highest reliability were equal to 1359, 662, 265 and 561 for HOLDK, HOLFR, JER and RDC, respectively. The number of QTL variants that led to the highest reliability was much larger for milk yield than for fat and protein yields, with, averaged across breeds, 1080 variants for milk yield, 564 for fat yield and 490 for protein yield.

**Table 4:** Scenarios with best reliability (r^2^) for each breed and trait

Breed	Trait	50 K^a^	Best scenario	n^b^	r^2^	∆^c^
HOLDK	Milk yield	0.44	MB-50 K + MBQTL10-25/1	3130	0.53	0.09
	Fat yield	0.48	MB-50 K + MBQTL20-25/1	424	0.58	0.10
	Protein yield	0.39	MB-50 K + MBQTL10-1/1	522	0.44	0.06
HOLFR	Milk yield	0.33	MB-50 K + MBQTL14-25/1	1046	0.41	0.08
	Fat yield	0.37	MB-50 K + MBQTL20-25/1	424	0.46	0.10
	Protein yield	0.37	MB-50 K + MBQTL14-25/10	517	0.44	0.06
JER	Milk yield	0.30	WB-50 K + MBQTL20-1/10	7	0.40	0.10
	Fat yield	0.16	MB-50 K + MBQTL10-10/10	775	0.20	0.04
	Protein yield	0.22	WB-50 K + MBQTL20-1/10	12	0.27	0.05
RDC	Milk yield	0.14	WB-50 K + MBQTL20-10/2	138	0.20	0.06
	Fat yield	0.11	WB-50 K + MBQTL14-10/2	635	0.19	0.07
	Protein yield	0.09	WB-50 K + MBQTL10-10/10	911	0.14	0.05

*HOLDK* Danish Holstein, *HOLFR* French Holstein, *JER* Jersey, *RDC* Danish Red

^a^Reliabilities when using 50 K SNPs

^b^Number of QTL variants included in the scenario

^c^Difference in reliability between the best scenario and that obtained with 50 K SNPs

**Fig. 1:**
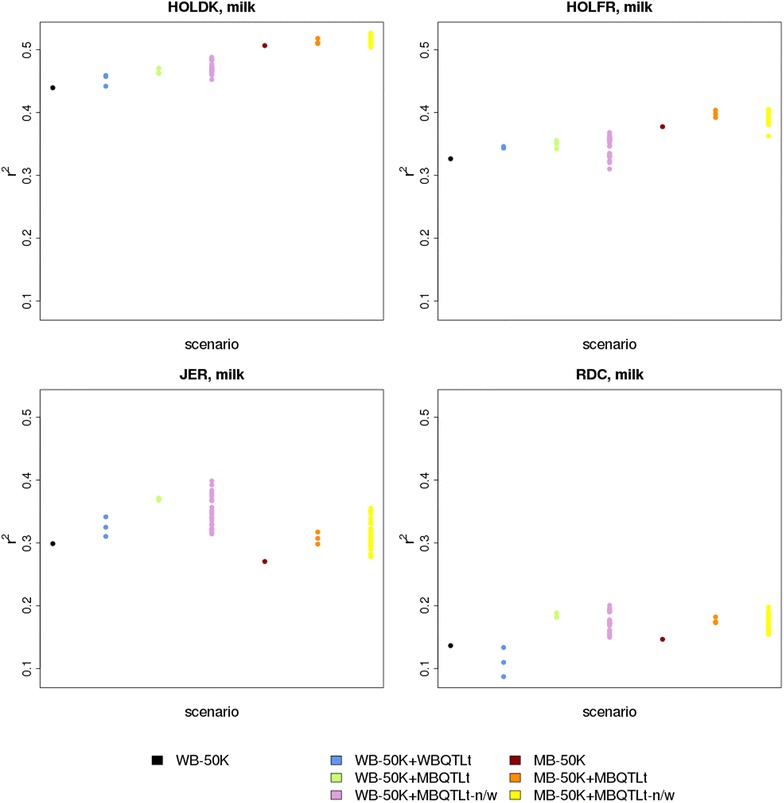
Reliabilities of genomic predictions in different scenarios for milk yield. HOLDK = Danish Holstein, HOLFR = French Holstein, JER = Jersey, RDC = Danish Red, WB-50 K = within-breed prediction using only 50 K SNPs, WB-50 K + WBQTLt = within-breed prediction using 50 K SNPs and a QTL component that contains variants selected with a p value below a threshold in a within-breed GWAS, WB-50 K + MBQTLt = within-breed prediction using 50 K SNPs and a QTL component that contains variants with a p value below a threshold in a multi-breed GWAS, WB-50 K + MBQTLt-n/w = within-breed prediction using 50 K SNPs and a QTL component that contains a limited number of variants within a QTL interval with a p value below a threshold in a multi-breed GWAS, MB-50 K = multi-breed prediction using 50 K SNPs, MB-50 K + MBQTLt = multi-breed prediction using 50 K SNPs and a QTL component that contains variants selected with a p value below a threshold in a multi-breed GWAS, MB-50 K + MBQTLt-n/w = multi-breed prediction using 50 K SNPs and a QTL component that contains a limited number of SNPs within a QTL interval with a p value below a threshold in a multi-breed GWAS

Increases in reliability of genomic predictions varied greatly depending on the set of QTL markers used compared with model WB-50 K. Figure 1 shows this variation among the scenarios investigated for milk yield, while the results for fat and protein yield are in Figure S1 (see Additional file 1: Figure S1).

Tables 5 and 6 compare reliabilities of genomic prediction between model WB-50 K and the other scenarios. Scenario WB-50 K-WBQTLt resulted in small increases up to 0.05 for the HOLDK and HOLFR populations, except for protein yield for HOLDK, which had a reliability that decreased by 0.01. For JER, reliabilities increased for milk and fat yield by 0.04 and 0.02, respectively, but no difference was found for protein yield. For RDC, only small differences were found, with decreases of 0.01 for fat and protein yield, and no difference for milk yield. The scenarios using QTL variants selected from the multi-breed GWAS showed increased reliabilities for all breeds. Larger increases were obtained when the number of variants per QTL region was limited. Averaged across breeds and traits, the differences in reliability between model WB-50 K and the other models using the QTL set that yielded the highest reliability (Δ_max_) were equal to 0.02, 0.03 and 0.05 for scenarios WB-50 K + WBQTLt, WB-50 K + MBQTLt, and WB-50 K + MBQTLt-n/w, respectively.

**Table 5:** Differences in reliability between the WB-50 K model and other models (Δ_max_) for within-breed prediction

Breed	Trait	WB-50 K + WBQTLt	WB-50 K + MBQTLt	WB-50 K + MBQTLt-n/w
HOLDK	Milk yield	0.02	0.03	0.05
	Fat yield	0.04	0.05	0.05
	Protein yield	−0.01	0.01	0.03
HOLFR	Milk yield	0.02	0.03	0.04
	Fat yield	0.05	0.05	0.05
	Protein yield	0.02	0.03	0.03
JER	Milk yield	0.04	0.07	0.10
	Fat yield	0.02	0.01	0.02
	Protein yield	0.00	0.02	0.05
RDC	Milk yield	0.00	0.05	0.06
	Fat yield	−0.01	0.03	0.07
	Protein yield	−0.01	0.01	0.05

The table provides only the Δ_max_, i.e. the difference (Δ) obtained with the QTL set that resulted in the highest reliability

The QTL component consisted of all the variants selected with a p value less than a threshold for the within-breed GWAS (WB-50 K + WBQTLt) and the multi-breed GWAS (WB-50 K + MBQTLt), or of a limited number of SNPs per QTL interval that were selected with p value less than a threshold for the multi-breed GWAS (WB-50 K + MBQTLt-n/w)

*HOLDK* Danish Holstein, *HOLFR* French Holstein, *JER* Jersey, *RDC* Danish Red

**Table 6:** Differences in reliability between the WB-50 K model and other models for multi-breed prediction

Breed^a^	Trait	MB-50 K (Δ^b^)	MB-50 K + MBQTLt (Δ_max_^c^)	MB-50 K + MBQTLt-n/w (Δ_max_^c^)
HOLDK	Milk	0.07	0.08	0.09
	Fat	0.06	0.09	0.10
	Protein	0.04	0.05	0.06
HOLFR	Milk	0.05	0.08	0.08
	Fat	0.06	0.09	0.10
	Protein	0.04	0.06	0.06
JER	Milk	−0.03	0.02	0.06
	Fat	0.01	−0.01	0.04
	Protein	−0.02	0.02	0.03
RDC	Milk	0.01	0.05	0.06
	Fat	0.02	0.06	0.05
	Protein	0.00	0.02	0.04

The model included one genetic component with all 50 K SNPs (MB-50 K), or an additional component that included either all SNPs selected with a p value less than a threshold for the multi-breed GWAS (MB-50 K + MBQTLt) or a limited number of variants per QTL interval selected with a p value less than a threshold for the multi-breed GWAS (MB-50 K + MBQTLt-n/w)

^a^HOLDK: Danish Holstein, HOLFR: French Holstein, JER: Jersey, RDC: Danish Red

^b^Differences in reliability between the reliability obtained with WB-50 K and the other models (Δ)

^c^For the models with a QTL component, the table provides only the Δ_max_, i.e. the Δ obtained with the QTL set that resulted in the highest reliability

Model MB-50 K led to substantial increases in reliability for HOLDK and HOLFR, only small differences for RDC, and a small decrease up to 0.03 for JER. For all breeds and traits, reliabilities were higher when variants selected from a multi-breed GWAS were used than when only 50 K SNPs were used. The best advantage was found when using the QTL variants for JER and RDC, while for HOLDK and HOLFR, the largest increases were obtained by combining the four populations. For most breeds and traits, reliabilities were higher when the number of QTL variants was limited than when all QTL variants were used. The largest difference between scenarios MB-50 K + MBQTLt and MB-50 K + MBQTLt-n/w was observed for JER for fat yield, with a Δ_max_ of −0.01 for the first and 0.04 for the latter model. Averaged across breeds and traits, Δ_max_ was equal to 0.03, 0.05 and 0.06 for the MB-50 K, MB-50 K + MBQTLt and MB-50 K + MBQTLt-n/w models, respectively.

### Bias

Regression coefficients of DRP on GEBV for all breeds and traits are in Table 7. In all scenarios, GEBV were overestimated, the bias being larger for JER and RDC than for HOLDK and HOLFR. Overall, using QTL variants for prediction had only a limited influence on the bias, with either an increase or a decrease in some scenarios compared to WB-50 K (see Table 7).

**Table 7:** Regression coefficients of DRP on GEBV for milk, fat and protein yield

Scenario	HOLDK	HOLFR	JER	RDC
*Milk yield*				
WB-50 K	0.83	0.72	0.67	0.71
WB-50 K + WBQTLt	0.82–0.83	0.72–0.72	0.62–0.71	0.46–0.56
WB-50 K + MBQTLt	0.83–0.84	0.70–0.73	0.70–0.70	0.67–0.70
WB-50 K + MBQTLt-n/w	0.83–0.86	0.68–0.74	0.68–0.78	0.61–0.72
MB-50 K	0.89	0.73	0.69	0.53
MB-50 K-MBQTLt	0.88–0.89	0.74–0.75	0.67–0.69	0.55–0.57
MB-50 K-MBQTLt-n/w	0.87–0.90	0.72–0.75	0.69–0.80	0.51–0.59
*Fat yield*				
WB-50 K	0.83	0.78	0.55	0.58
WB-50 K + WBQTLt	0.82–0.82	0.79–0.81	0.49–0.58	0.45–0.49
WB-50 K + MBQTLt	0.82–0.82	0.80–0.80	0.59–0.59	0.52–0.54
WB-50 K + MBQTLt-n/w	0.79–0.82	0.78–0.82	0.50–0.61	0.49–0.62
MB-50 K	0.81	0.87	0.57	0.40
MB-50 K-MBQTLt	0.81–0.81	0.87–0.88	0.52–0.56	0.43–0.46
MB-50 K-MBQTLt-n/w	0.80–0.82	0.87–0.90	0.47–0.62	0.38–0.46
*Protein yield*				
WB-50 K	0.75	0.74	0.61	0.57
WB-50 K + WBQTLt	0.72–0.74	0.74–0.75	0.47–0.60	0.41–0.50
WB-50 K + MBQTLt	0.73–0.75	0.75–0.76	0.60–0.61	0.55–0.59
WB-50 K + MBQTLt-n/w	0.73–0.77	0.72–0.76	0.57–0.64	0.55–0.72
MB-50 K	0.80	0.77	0.60	0.43
MB-50 K-MBQTLt	0.78–0.80	0.78–0.78	0.63–0.64	0.44–0.49
MB-50 K-MBQTLt-n/w	0.79–0.82	0.76–0.79	0.62–0.68	0.36–0.54

*HOLDK* Danish Holstein, *HOLFR* French Holstein, *JER* Jersey, *RDC* Danish Red

### Influence of the number of QTL markers on reliability of genomic prediction and on the variance explained by QTL markers

**Fig. 2:**
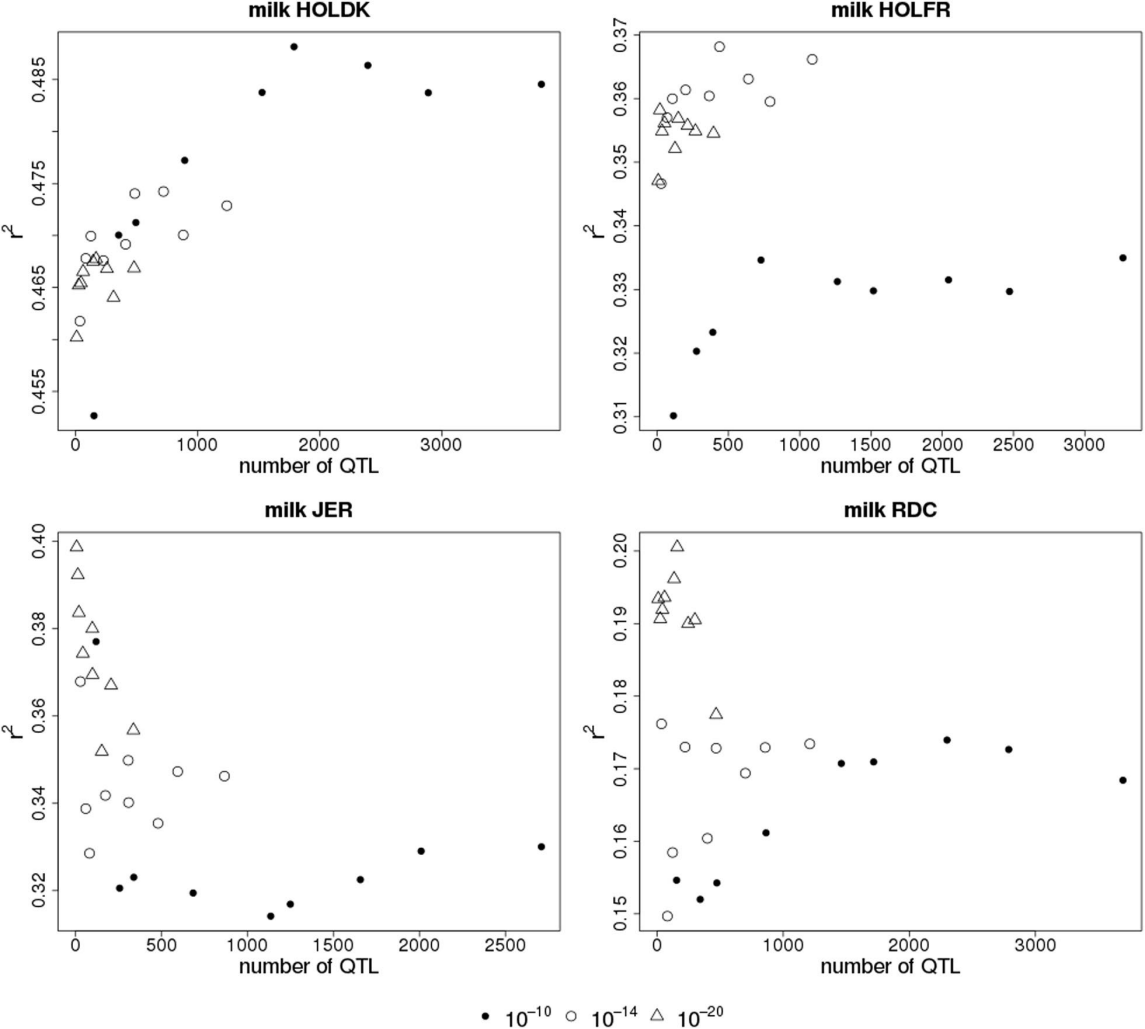
Reliability of genomic predictions according to number of QTL markers for milk yield. HOLDK = Danish Holstein, HOLFR = French Holstein, JER = Jersey and RDC = Danish Red. Reliabilities are shown for within-breed prediction using 50 K and QTL components that contain a limited number of variants within a QTL interval selected with a p value below a threshold of 10^−10^ (*closed circles*), 10^−14^ (*open circles*) or 10^−20^ (*triangles*) in a multi-breed GWAS

The number of selected QTL markers varied markedly between scenarios. Figure 2 shows reliabilities of genomic predictions according to number of QTL markers used for the WB-50 K-MBQTLt-n/w scenarios for milk yield. Results for fat and protein yield are in Figure S2 (see Additional file 2: Figure S2). Although reliability of genomic prediction depended on the number of QTL variants used, there were no clear peaks, but overall reliabilities were highest when a relatively small number of QTL variants was used.

**Fig. 3:**
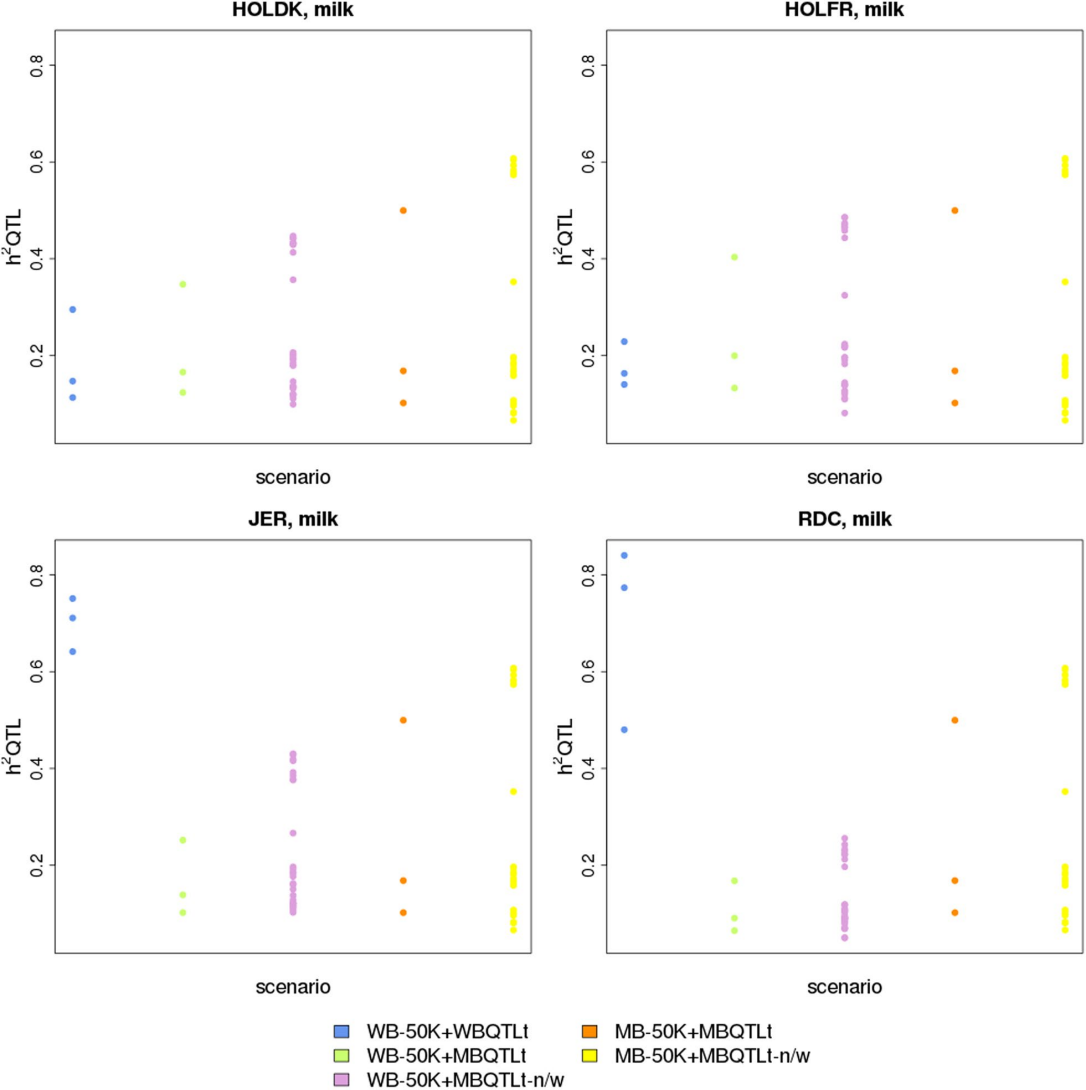
Heritabilities of the QTL component (h^2^ QTL) in different scenarios for milk yield. HOLDK = Danish Holstein, HOLFR = French Holstein, JER = Jersey, RDC = Danish Red, WB-50 K = within-breed prediction using only 50 K SNPs, WB-50 K + WBQTLt = within-breed prediction using 50 K SNPs and a QTL component that contains variants selected with a p value below a threshold in a within-breed GWAS, WB-50 K + MBQTLt = within-breed prediction using 50 K SNPs and a QTL component that contains variants with a p value below a threshold in a multi-breed GWAS, WB-50 K + MBQTLt-n/w = within-breed prediction using 50 K SNPs and a QTL component that contains a limited number of variants within a QTL interval with a p value below a threshold in a multi-breed GWAS, MB-50 K = multi-breed prediction using 50 K SNPs, MB-50 K + MBQTLt = multi-breed prediction using 50 K SNPs and a QTL component that contains variants selected with a p value below a threshold in a multi-breed GWAS, MB-50 K + MBQTLt-n/w = multi-breed prediction using 50 K SNPs and a QTL component that contains a limited number of SNPs within a QTL interval with a p value below a threshold in a multi-breed GWAS

The proportion of variance explained by the QTL component ($h_{QTL}^{2}$) varied greatly between scenarios, as shown in Fig. 3 for milk yield, and Figure S3 (see Additional file 3: Figure S3) for fat and protein yield. The $h_{QTL}^{2}$ obtained in the WB-50 K + WBQTLt scenarios was much larger for the JER and RDC breeds than for the HOLDK and HOLFR populations. For HOLDK and HOLFR, regardless of the scenarios using a QTL component selected from the multi-breed GWAS, $h_{QTL}^{2}$ was either larger or smaller than that obtained in scenario WB-50 K + WBQTLt, depending on the criteria that were applied for QTL selection, while for JER and RDC, $h_{QTL}^{2}$ was almost always substantially larger in scenarios WB-50 K + WBQTLt.

**Fig. 4:**
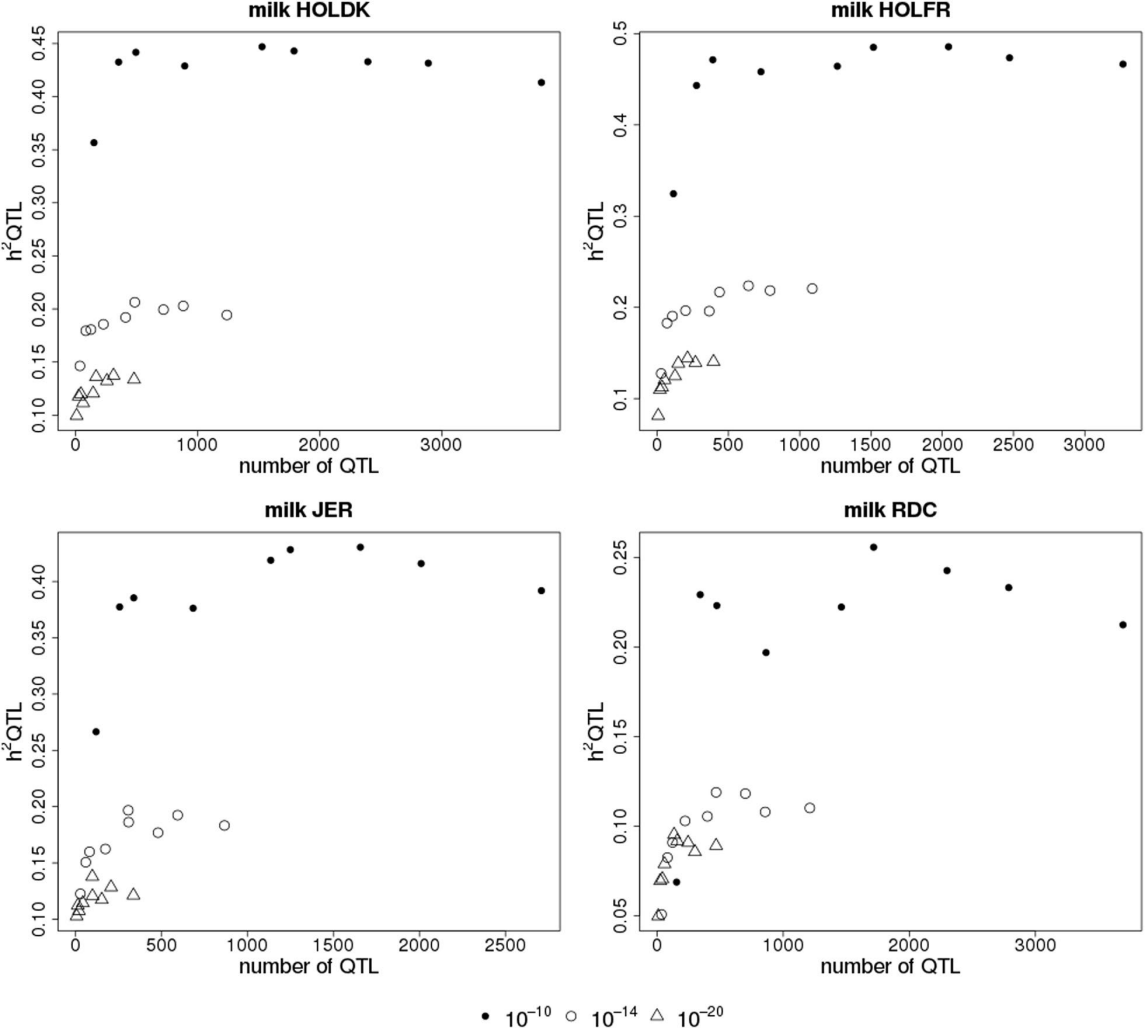
Heritabilities of QTL (h^2^ QTL) according to number of QTL markers for milk yield. HOLDK = Danish Holstein, HOLFR = French Holstein, JER = Jersey and RDC = Danish Red. Reliabilities are shown for within-breed prediction using 50 K and QTL components that contain a limited number of variants within a QTL interval selected with a p value below a threshold of 10^−10^ (*closed circles*), 10^−14^ (*open circles*) or 10^−20^ (*triangles*) in a multi-breed GWAS

For all breeds, $h_{QTL}^{2}$ was influenced by the number of QTL variants used in the QTL component, as shown in Fig. 4 for the WB-50 K + MBQTLt-n/w scenarios for milk yield, and Figure S4 (see Additional file 4: Figure S4) for fat and protein yield. In scenarios WB-50 K + MBQTLt, the number of selected QTL variants depended solely on the threshold applied for QTL selection, and $h_{QTL}^{2}$ increased approximately linearly with the number of QTL variants (results not shown). In the sets used for scenarios WB-50 K + MBQTLt-n/w, $h_{QTL}^{2}$ was larger for the sets with a lower selection threshold and thus a larger number of QTL. For scenarios WB-50 K + MBQTLt-n/w in which the same threshold was applied, $h_{QTL}^{2}$ fluctuated a lot without necessarily increasing if a larger number of QTL variants was used. Sets used in scenarios MBQTLt-n/w and MBQTLt led to similar $h_{QTL}^{2}$, while MBQTLt-n/w included much fewer QTL variants than MBQTLt (results not shown).

### Genomic correlations between breeds

**Fig. 5:**
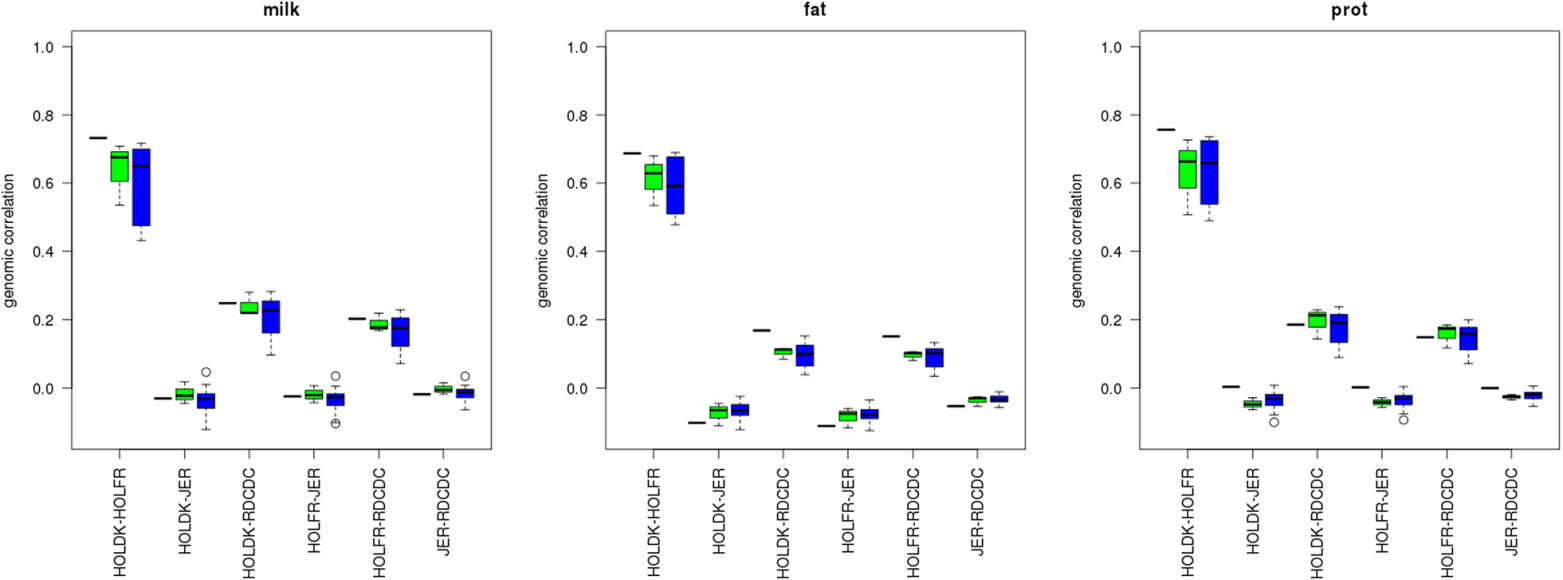
Genomic correlations of 50 K SNP effects between breeds. HOLDK = Danish Holstein, HOLFR = French Holstein, JER = Jersey, RDC = Danish Red, scenarios using only 50 K SNPs (*black*), 50 K SNPs and a QTL component with all (*green*) or a limited number of variants per QTL region (*blue*) that were selected from a multi-breed GWAS

For the multi-trait models, genomic correlations between the same traits in different breeds were estimated. Figure 5 shows the genomic correlations using the 50 K component in the MT-50 K, MT-50 K + MBQTLt and MT-50 K + MBQTLt-n/w models. Genomic correlations of the 50 K component ranged from 0.43 to 0.76 between HOLDK and HOLFR, from 0.03 to 0.28 between HOLDK or HOLFR and RDC, and from −0.12 to 0.05 between JER and any other breed.

**Fig. 6:**
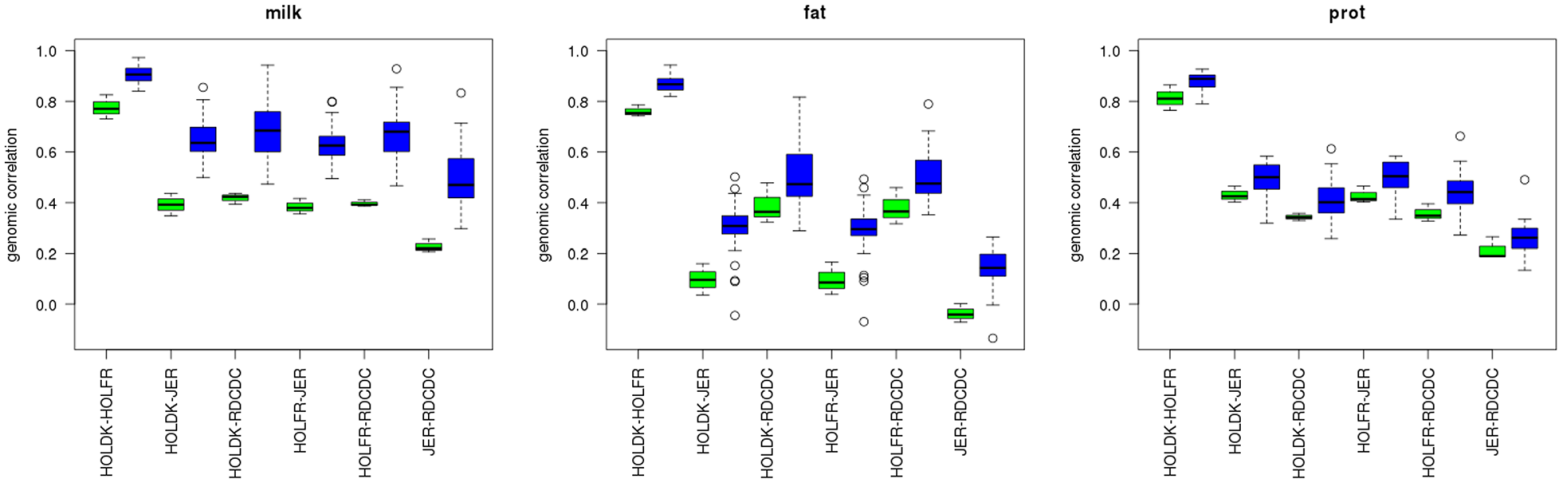
Genomic correlations of QTL effects between breeds. HOLDK = Danish Holstein, HOLFR = French Holstein, JER = Jersey, RDC = Danish Red, scenarios using only 50 K SNPs (*black*), 50 K SNPs and a QTL component with all (*green*) or a limited number of variants per QTL region (*blue*) that were selected from a multi-breed GWAS

Genomic correlations that were computed by using the QTL component and the MT-50 K + MBQTLt and MT-50 K + MBQTLt-n/w scenarios are in Fig. 6. All genomic correlations were larger when the QTL component was used than when the 50 K component was used. The largest correlations were obtained with the MT-50 K + MBQTLt-n/w scenarios. Genomic correlations between HOLDK and HOLFR ranged from 0.73 to 0.86 for MT-50 K + MBQTt-n/w and from 0.79 to 0.97 for MT-50 K + MBQTLt-n/w. Between HOLDK or HOLFR and RDC, genomic correlations that were computed by using the QTL component ranged from 0.32 to 0.48 for MT-50 K + MBQTLt, and from 0.26 to 0.94 with the MT-50 K + MBQTLt-n/w and between JER and the other breeds, the lowest correlations were found for fat yield (ranging from −0.07 to 0.17 for MT-50 K + MBQTLt and from −0.13 to 0.50 for MT-50 K + MBQTLt-n/w), while for milk and protein yield, they were always positive (ranging from 0.19 to 0.46 for MT-50 K + MBQTLt and from 0.13 to 0.86 for MT-50 K + MBQTLt-n/w).

Posterior standard deviations of the genomic correlations are in Table S1 (see Additional file 5: Table S1); they ranged from 0.01 to 0.33 when the QTL components were used and from 0.03 to 0.17 when the 50 K component was used. Standard deviations were smallest between the two Holstein populations (on average 0.02 for the QTL components and 0.05 for the 50 K component), and larger for any other breed combination (on average 0.16 for the QTL components and 0.11 for the 50 K component).

## Discussion

The advantage of whole-genome sequence data is that they include causative mutations. However, some causative mutations may be absent, for example because of partial variant calling that does not consider structural variants, and because some variants may be filtered out due to poor sequencing and imputation quality. Furthermore, the locations of the causative mutations present in the data are unknown. Thus, we attempted to identify variants that were in high LD with the causative mutations based on GWAS data. Using QTL variants that were selected from a multi-breed GWAS for within-breed prediction resulted in substantial increases in the reliability of genomic predictions for all breeds and traits compared to a 50 K within-breed model. While the reliability of multi-breed prediction increased when QTL markers were used rather than only 50 K SNPs, multi-breed reliabilities were very similar to within-breed reliabilities when markers in the QTL component were chosen based on multi-breed GWAS data.

Increases in reliabilities observed for the two Holstein populations when within-breed QTL variants were used were in the range of those reported by Brøndum et al. [17]. In RDC, inclusion of within-breed QTL variants decreased the reliability of genomic predictions. This can be explained by the large difference in population size used for the GWAS that was performed to select QTL variants. For the within-breed GWAS, each Holstein population included about 5000 individuals, while the JER and RDC populations each included less than 1000 individuals, which results in much more detection power for HOLFR and HOLDK than for JER and RDC. Thus, selection of variants for JER and RDC is likely less reliable, and they add noise rather than information on the causative mutations, which results in a reduced reliabilities of genomic predictions.

We expected that selected variants from the multi-breed GWAS would be beneficial mainly for multi-breed prediction, but not necessarily for within-breed prediction for JER and RDC, since the multi-breed GWAS was dominated by Holstein animals. Within-breed prediction using variants selected from the multi-breed GWAS did, however, increase reliabilities of genomic predictions for all breeds, including JER and RDC. Our findings confirm those from other studies [11, 28], which showed that a multi-breed GWAS results in more accurate QTL mapping than a within-breed GWAS. While adding QTL variants selected from the multi-breed GWAS resulted in increased reliabilities for all breeds and traits, they were highly sensitive to the choice of the QTL markers. This is in line with results from a study by Ober et al. [19], who used variants that were selected from a GWAS for genomic prediction of quantitative traits in *Drosophila*. They showed that accuracy of genomic prediction varied strongly with the threshold used to select prediction variants. In our study, the highest reliabilities were always obtained when the number of QTL variants per region was limited. This confirms our expectation that a restricted number of prediction markers per QTL interval leads to higher reliabilities than selecting a larger number of markers. Although the most significant variant in a GWAS is not necessarily the causative mutation, variants near the peak are more likely to be in high LD with the causative mutation, while variants further away are likely to be in lower LD and therefore, introduce more noise in the prediction. Therefore, restricting the number of variants per QTL interval resulted in higher reliabilities than selecting all variants with p values below a threshold. The optimal filtering, regarding both p value and restriction of variants per region, depended on breed and trait. For the JER breed, reliabilities of genomic predictions were highest with much fewer variants than for the other breeds. Again, this can be explained by the short distances over which LD is conserved across breeds. The multi-breed GWAS was dominated by Holstein animals, but also used the data from other breeds. Therefore, the variants that are in LD with the causative mutations in both Holstein populations and the other breeds, are likely to be among the variants with the most significant p values, while variants further away from the peak, may only be in LD with the causative mutation in the Holstein populations.

The variance explained by the QTL component varied strongly between breeds, traits and sets of prediction markers. Although for JER and RDC the WB-50 K + WBQTLt scenarios led to similar or lower reliabilities compared to model WB-50 K, the QTL markers used in those scenarios did explain a substantially larger part of the total genetic variance than the other sets of prediction markers. In these scenarios, the QTL markers may estimate a polygenic effect rather than accurately estimate the effects of the largest QTL, but are actually less accurate in capturing the polygenic effect than WB-50 K, and thereby, reducing the reliability of genomic predictions. While the QTL markers used in the WB-50 K + MBQTLt-n/w scenarios only explained a small part of the total genetic variance, their use resulted in large increases in the reliability of genomic predictions for JER and RDC.

The advantage of having a second genetic component with QTL variants in the SNP BLUP model could be due to some variants having a larger effect rather than to the specific variants being included in the QTL component. If this is the case, the advantage of the QTL component will be smaller with a mixture model. We tested the WB-50 K, WB-50 K + MBQTLt, and WB-50 K + MBQTLt-n/w scenarios also with a Bayesian mixture model that fitted two mixture distributions for the 50 K SNPs, and two different mixture distributions for the QTL markers. Reliabilities of genomic predictions obtained with the Bayesian mixture model and the SNP BLUP model were similar and increases obtained by adding a multi-breed QTL component were within the same range (results not shown).

Combining all populations in the multi-breed models led to higher reliabilities than within-breed prediction only for HOLDK and HOLFR, which is not surprising, since the Holstein reference population was approximately doubled by combining the two HOLDK and HOLFR populations. While the use of a multi-breed population and sequence information is valuable in pinpointing the location of variants that are in close LD with the causative variants, using these variants for multi-breed prediction is, however, straightforward. Variant effects can differ between breeds and multi-breed prediction models can carry noise from a large population to smaller populations. This confirms our expectation, that when combining data from multiple breeds, the single-trait models are suitable for closely related populations, but not for more distantly related breeds, because they assume equal variant effects across populations.

The multi-trait models allow the estimation of genomic correlations of marker effects across traits. The correlations obtained with the 50 K SNPs confirmed the relatedness between the different populations: while the Holstein populations are highly related and the RDC and Holstein populations are moderately related, genomic correlations between the JER breed and either of the other breeds are approximately 0. With such correlations, it is not surprising that with model MB-50 K, which assumes similar marker effects for all breeds, reliability of genomic predictions did not increase for RDC and decreased for JER. However, genomic correlations estimated for the multi-breed QTL components were moderate to high, even between JER and the other breeds, indicating that the multi-breed QTL components did contain variants that were associated with QTL segregating across breeds. The fact that higher genomic correlations were obtained in the MT-50 K + MBQTLt-n/w scenarios than in the MT-50 K + MBQTLt scenarios confirms that stricter selection criteria result in the selection of variants that are closely located to the causative mutations. However with such high across-breed correlations, it is surprising that the use of a multi-breed reference population yielded no advantage for JER and RDC. This is probably due to the low across-breed correlations of the 50 K SNPs.

Although the multi-trait model allowed the 50 K SNPs and the QTL markers to have different genomic correlations, reliabilities of genomic predictions were similar to those obtained in the WB-50 K + MBQTLt and WB-50 K + MBQTLt-n/i scenarios (results not shown).

To take advantage of the highly correlated multi-breed QTL effects, without having to overcome the noise introduced by the 50 K SNPs, a model that includes a multi-breed QTL component but only within-breed 50 K components may result in increased reliabilities compared to within-breed prediction. Porto-Neto et al. [18] showed that to improve across-breed prediction, it is important to select variants that are highly correlated across breeds. In their study, variants were selected from a GWAS within Brahman and Tropical composite cattle. Variants with effects in the same direction in both breeds resulted in increased across-breed reliabilities of genomic predictions and high genomic correlations, while variants with opposite effects decreased reliabilities and resulted in negative genomic correlations. By fitting separate within- and multi-breed genomic relationship matrices, Khansefid et al. [36] reported increases in accuracy for some traits compared to a model using only within-breed relationships.

None of the sets of prediction markers used here yielded the highest reliability for all breeds. Although such a set would be ideal, it might not be realistic. Variants that play an important role in one breed, could actually introduce noise in another breed. Furthermore, QTL properties such as allele frequencies influence accuracy [37], and can differ between breeds and traits. Rather than testing a large number of prediction sets to find the optimal set for each breed and trait, as was done in this study, a multi-trait Bayesian variable selection model as described by Janss [38] could potentially select the most adequate variants for each breed.

Several studies have shown that, using full sequence data directly for genomic prediction, rather than preselecting variants, does not improve prediction reliability [14, 15]. Our results show that both prediction reliability and genomic correlations across populations and breeds are highly sensitive to the choice of the prediction markers. Full sequence data is likely to result in similar genomic relationships and correlations as genome-wide SNPs, and is therefore unlikely to improve prediction reliability. Bayesian variable selection models allow for heterogeneous variances and could potentially exploit the presence of causative mutations in the sequence data by assigning non-zero effects to variants that are close to the causative mutations, and zero effects to all other variants. However, in practice Van Binsbergen et al. [15] found no increase in prediction reliability using full sequence data compared to SNPs, even with a Bayesian variable selection model. A potential explanation for the lack of improvement in prediction reliability could be that the number of SNPs is much larger than the number of individuals. The number of SNPs can be significantly reduced by preselecting SNPs based on their functional annotations, for example by using only SNPs located within genes. By doing this, Hayes et al. [39] reported a 2% increase in prediction accuracy in Holstein cattle, averaged over production traits. Erbe et al. [9] showed that the use of variants from the transcribed regions of the genome resulted in higher accuracy for across-breed prediction compared to prediction based on 50 K genotypes. Selection of variants based on functional annotations could also be used to refine the selection of variants per interval by giving preference to variants located in genes rather than only selecting variants based on their statistical association detected in a GWAS.

Selecting prediction variants based on their association with a trait could result in prediction bias. While there was bias in all our results, the inclusion of QTL markers did not consistently increase the bias, i.e. it increased or decreased depending on the set of QTL markers used. Regression coefficients were always less than 1, which indicates that the GEBV were overestimated for the test animals. This may be due to inflated GEBV and strong selection of individuals in the test population for the traits in the analyses. Furthermore, this effect of selection was increased by the fact that the sires used for prediction were removed from the reference population.

While some sets of QTL markers resulted in substantial increases in prediction reliability for the populations that were tested in our study, this may not be true for other populations. The optimal set of prediction markers differed between populations, and the sets that we identified are not necessarily the best sets for other populations. Furthermore, we studied milk traits for which few QTL are known to have large effects. Increasing prediction reliability by adding sequence variants is likely to be more challenging for more polygenic traits. Brøndum et al. [17] found smaller increases in prediction reliability for mastitis and fertility than for production traits. Our results do not provide a list of markers that increase prediction, but they do demonstrate that sequence variants can potentially increase prediction reliability. In our analyses, we tested a large number of prediction sets, which is not practical for routine genomic evaluation. An alternative could be to make a less stringent selection of prediction markers, but subsequently use a more sophisticated prediction model, that allows marker effects to differ between breeds and traits. Further research is required to develop a more practical way to exploit sequence data for genomic prediction.

## Conclusions

Prediction reliability increased substantially for all breeds and traits when sequence variants selected from a GWAS were used for genomic prediction. Even for within-breed prediction, a multi-breed GWAS was more efficient in identifying variants that increase prediction reliability than within-breed GWAS. Prediction reliabilities were highly sensitive to the choice of prediction markers, and limiting the number of variants per QTL region led to higher prediction reliabilities than selecting them on the basis of a p value threshold. While the highest prediction reliabilities were obtained within breed, multi-breed prediction reliabilities were higher than multi-breed prediction reliabilities when using only 50 K SNPs, and across breed genomic correlations of QTL variants were much higher than those obtained at 50 K SNPs. Our results show that sequence data can potentially increase reliabilities of genomic predictions, if the proper variants are used, which is more likely if they are selected from a multi-breed GWAS.

## Additional files

**Additional file 1: Figure S1.** Reliabilities of genomic predictions in different scenarios for fat and protein yield. HOLDK = Danish Holstein, HOLFR = French Holstein, JER = Jersey, RDC = Danish Red, WB-50 K = within-breed prediction using only 50 K SNPs, WB-50 K + WBQTLt = within-breed prediction using 50 K SNPs and a QTL component that contains variants selected with a p value below a threshold in a within-breed GWAS, WB-50 K + MBQTLt = within-breed prediction using 50 K SNPs and a QTL component that contains variants with a p value below a threshold in a multi-breed GWAS, WB-50 K + MBQTLt-n/w = within-breed prediction using 50 K SNPs and a QTL component that contains a limited number of variants within a QTL interval with a p value below a threshold in a multi-breed GWAS, MB-50 K = multi-breed prediction using 50 K SNPs, MB-50 K + MBQTLt = multi-breed prediction using 50 K SNPs and a QTL component that contains variants selected with a p value below a threshold in a multi-breed GWAS, MB-50 K + MBQTLt-n/w = multi-breed prediction using 50 K SNPs and a QTL component that contains a limited number of SNPs within a QTL interval with a p value below a threshold in a multi-breed GWAS.

**Additional file 2: Figure S2.** Reliabilities of genomic predictions according to number of QTL markers for fat and protein yield. HOLDK = Danish Holstein, HOLFR = French Holstein, JER = Jersey and RDC = Danish Red. Reliabilities are shown for within breed prediction using 50 K and QTL components containing a restricted number of markers in a QTL interval with a p value below a threshold of 10^−10^ (closed circles), 10^−14^ (open circles) or 10^−20^ (triangles) in a multi breed GWAS.

**Additional file 3: Figure S3.** Heritabilities of the QTL component (h^2^ QTL) in different scenarios for fat and protein yield. HOLDK = Danish Holstein, HOLFR = French Holstein, JER = Jersey, RDC = Danish Red, WB-50 K = within-breed prediction using only 50 K markers, WB-50 K + WBQTLt = within breed prediction using 50 K markers and a QTL component containing markers with a p value below a threshold in a within breed GWAS, WB-50 K + MBQTLt = within breed prediction using 50 K markers and a QTL component containing markers with a p value below a threshold in a multi breed GWAS, WB-50 K + MBQTLt-n/w = within breed prediction using 50 K markers and a QTL component containing a restricted number of markers in a QTL interval with a p value below a threshold in a multi breed GWAS, MB-50 K = multi breed prediction using 50 K markers, MB-50 K + MBQTLt = multi breed prediction using 50 K markers and a QTL component containing markers with a p value below a threshold in a multi breed GWAS, MB-50 K + MBQTLt-n/w = multi breed prediction using 50 K markers and a QTL component containing a restricted number of markers in a QTL interval with a p value below a threshold in a multi breed GWAS.

**Additional file 4: Figure S4.** Heritabilities of QTL (h^2^ QTL) according to number of QTL markers for fat and protein yield. HOLDK = Danish Holstein, HOLFR = French Holstein, JER = Jersey and RDC = Danish Red. Reliabilities are shown for within-breed prediction using 50 K and QTL components containing a restricted number of markers in a QTL interval with a p value below a threshold of 10^−10^ (closed circles), 10^−14^ (open circles) or 10^−20^ (triangles) in a multi breed GWAS.

**Additional file 5: Table S1.** Posterior standard deviations of genomic correlations between breeds. Average standard deviation across scenarios followed by minimum and maximum standard deviation, for the 50 K component (σ_50K_), QTL component where QTL markers are selected based on their p value (σ_MBQTLt_) and QTL component where the maximum number of markers per QTL window is restricted (σ_MBQTLt-n/w_), HOLDK = Danish Holstein, HOLFR = French Holstein, JER = Jersey and RDC = Danish Red.
