# Supplementary figures and images for: Sequence variants selected from a multi-breed GWAS can improve the reliability of genomic predictions in dairy cattle
Source: Genet Sel Evol. 2016 Nov 4;48:83. doi: 10.1186/s12711-016-0259-0 (PMC5095991; doi:10.1186/s12711-016-0259-0)

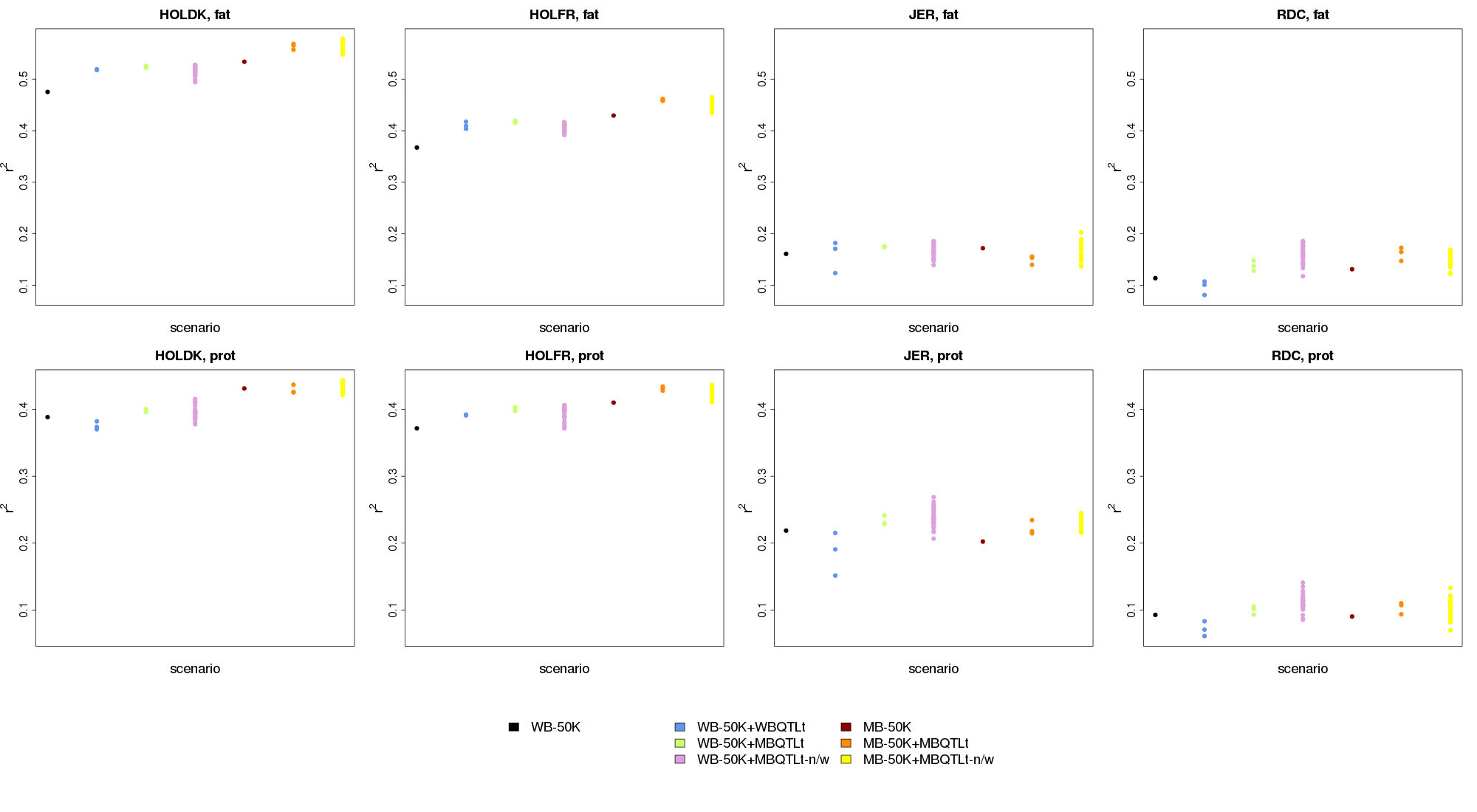

Supplement: Supplementary file 1 — Additional file 1: Figure S1. Reliabilities of genomic predictions in different scenarios for fat and protein yield. HOLDK = Danish Holstein, HOLFR = French Holstein, JER = Jersey, RDC = Danish Red, WB-50 K = within-breed prediction using only 50 K SNPs, WB-50 K + WBQTLt = within-breed prediction using 50 K SNPs and a QTL component that contains variants selected with a p value below a threshold in a within-breed GWAS, WB-50 K + MBQTLt = within-breed prediction using 50 K SNPs and a QTL component that contains variants with a p value below a threshold in a multi-breed GWAS, WB-50 K + MBQTLt-n/w = within-breed prediction using 50 K SNPs and a QTL component that contains a limited number of variants within a QTL interval with a p value below a threshold in a multi-breed GWAS, MB-50 K = multi-breed prediction using 50 K SNPs, MB-50 K + MBQTLt = multi-breed prediction using 50 K SNPs and a QTL component that contains variants selected with a p value below a threshold in a multi-breed GWAS, MB-50 K + MBQTLt-n/w = multi-breed prediction using 50 K SNPs and a QTL component that contains a limited number of SNPs within a QTL interval with a p value below a threshold in a multi-breed GWAS. [file 12711_2016_259_MOESM1_ESM.png]

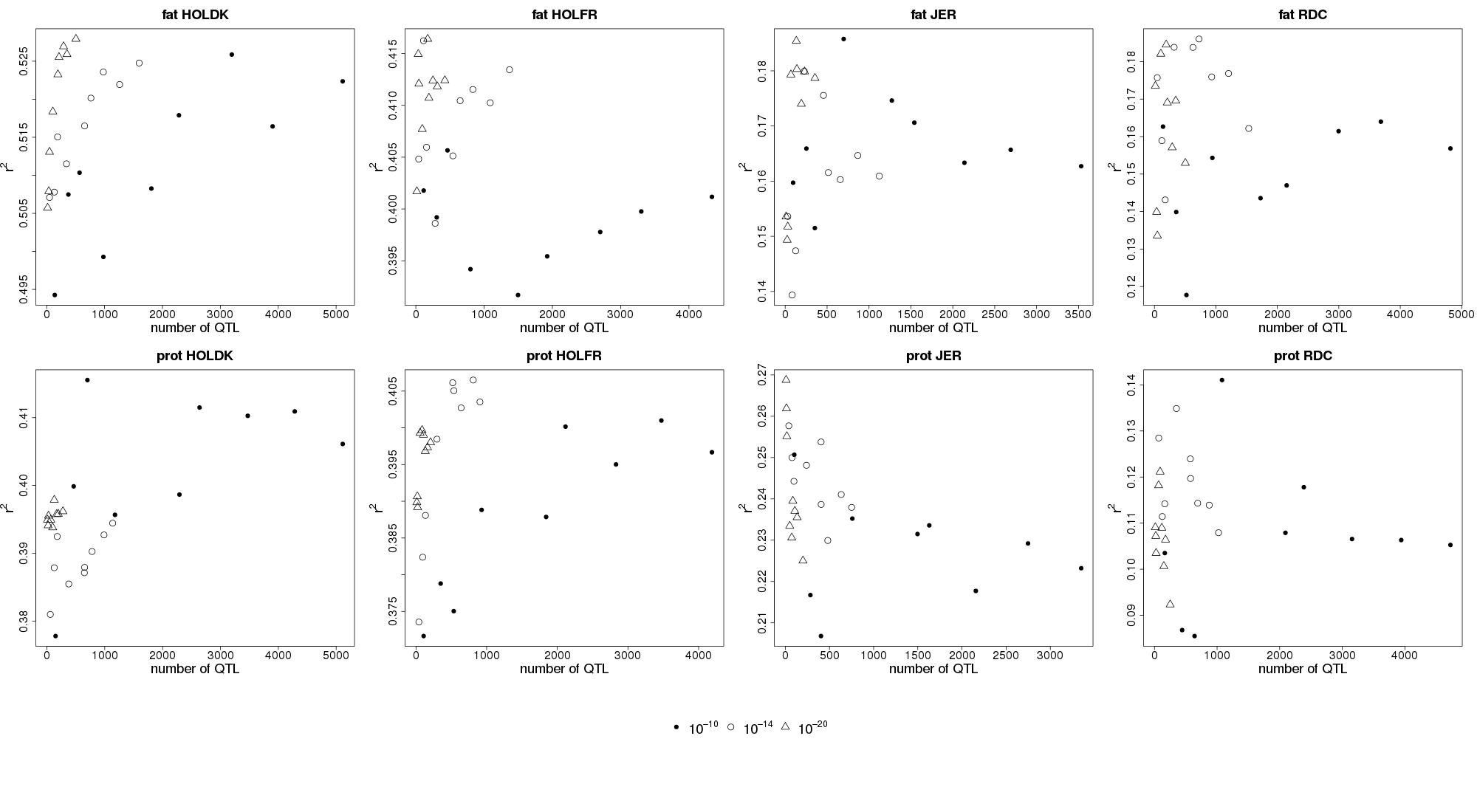

Supplement: Supplementary file 2 — Additional file 2: Figure S2. Reliabilities of genomic predictions according to number of QTL markers for fat and protein yield. HOLDK = Danish Holstein, HOLFR = French Holstein, JER = Jersey and RDC = Danish Red. Reliabilities are shown for within breed prediction using 50 K and QTL components containing a restricted number of markers in a QTL interval with a p value below a threshold of 10−10 (closed circles), 10−14 (open circles) or 10−20 (triangles) in a multi breed GWAS. [file 12711_2016_259_MOESM2_ESM.png]

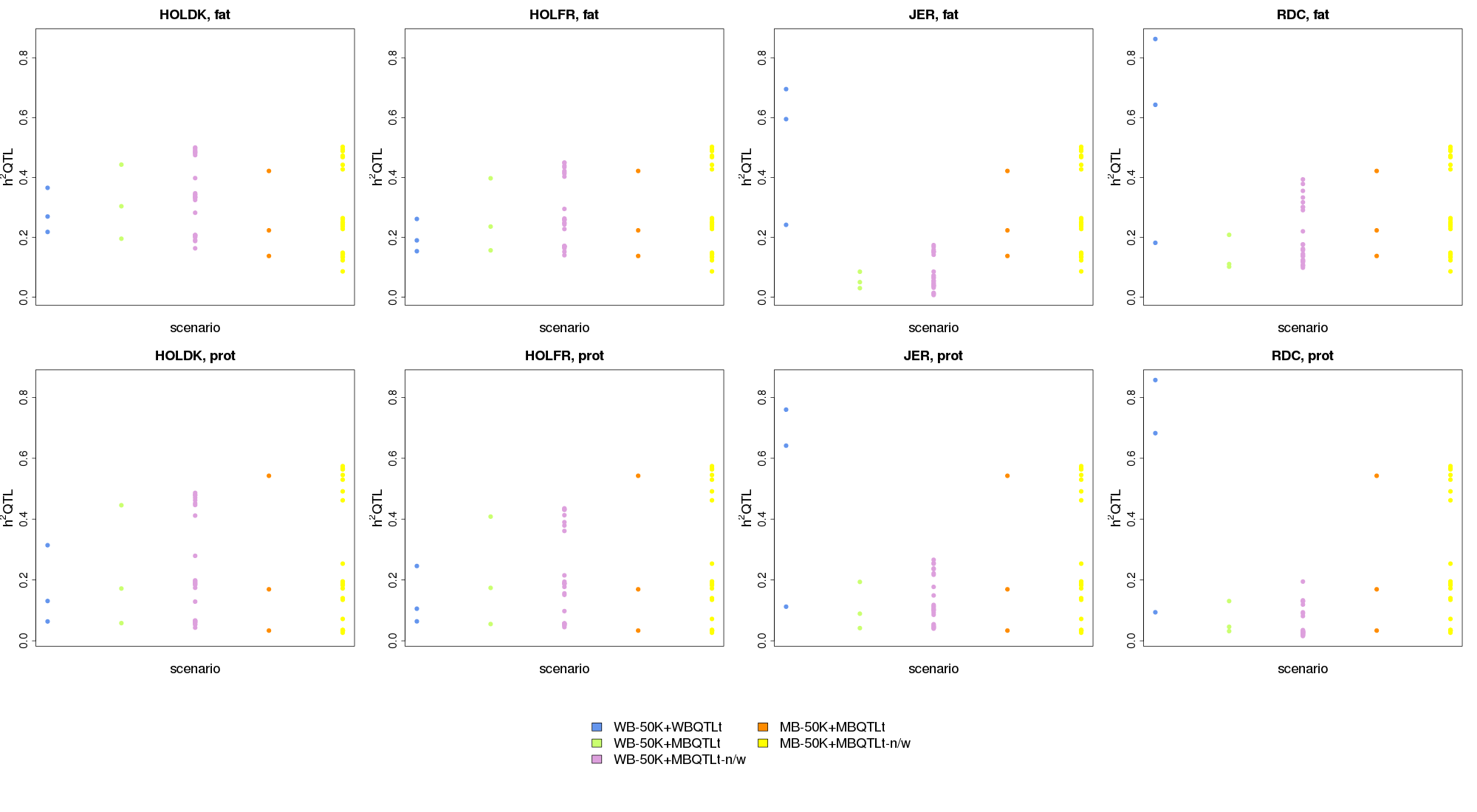

Supplement: Supplementary file 3 — Additional file 3: Figure S3. Heritabilities of the QTL component (h2 QTL) in different scenarios for fat and protein yield. HOLDK = Danish Holstein, HOLFR = French Holstein, JER = Jersey, RDC = Danish Red, WB-50 K = within-breed prediction using only 50 K markers, WB-50 K + WBQTLt = within breed prediction using 50 K markers and a QTL component containing markers with a p value below a threshold in a within breed GWAS, WB-50 K + MBQTLt = within breed prediction using 50 K markers and a QTL component containing markers with a p value below a threshold in a multi breed GWAS, WB-50 K + MBQTLt-n/w = within breed prediction using 50 K markers and a QTL component containing a restricted number of markers in a QTL interval with a p value below a threshold in a multi breed GWAS, MB-50 K = multi breed prediction using 50 K markers, MB-50 K + MBQTLt = multi breed prediction using 50 K markers and a QTL component containing markers with a p value below a threshold in a multi breed GWAS, MB-50 K + MBQTLt-n/w = multi breed prediction using 50 K markers and a QTL component containing a restricted number of markers in a QTL interval with a p value below a threshold in a multi breed GWAS. [file 12711_2016_259_MOESM3_ESM.png]

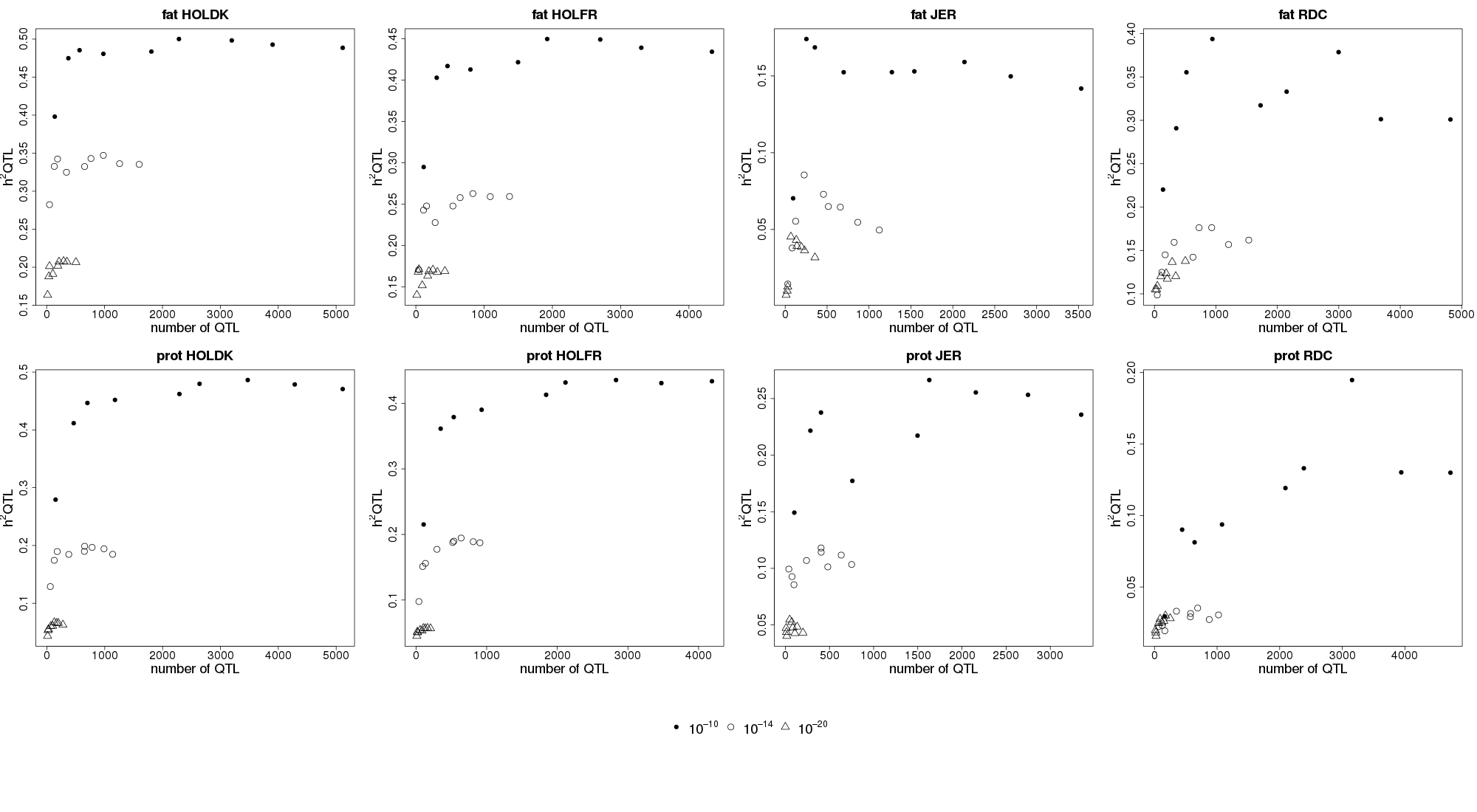

Supplement: Supplementary file 4 — Additional file 4: Figure S4. Heritabilities of QTL (h2 QTL) according to number of QTL markers for fat and protein yield. HOLDK = Danish Holstein, HOLFR = French Holstein, JER = Jersey and RDC = Danish Red. Reliabilities are shown for within-breed prediction using 50 K and QTL components containing a restricted number of markers in a QTL interval with a p value below a threshold of 10−10 (closed circles), 10−14 (open circles) or 10−20 (triangles) in a multi breed GWAS. [file 12711_2016_259_MOESM4_ESM.png]
