# Supplementary material for: Sequence variants selected from a multi-breed GWAS can improve the reliability of genomic predictions in dairy cattle
Source: Genet Sel Evol. 2016 Nov 4;48:83. doi: 10.1186/s12711-016-0259-0 (PMC5095991; doi:10.1186/s12711-016-0259-0)
Supplement: Supplementary file 5 — Additional file 5: Table S1. Posterior standard deviations of genomic correlations between breeds. Average standard deviation across scenarios followed by minimum and maximum standard deviation, for the 50 K component (σ50K), QTL component where QTL markers are selected based on their p value (σMBQTLt) and QTL component where the maximum number of markers per QTL window is restricted (σMBQTLt-n/w), HOLDK = Danish Holstein, HOLFR = French Holstein, JER = Jersey and RDC = Danish Red. [file 12711_2016_259_MOESM5_ESM.docx]

| **Trait** | **Breeds** | **σ_50K_** | **σ_MBQTLt_** | **σ_MBQTLt-n/w_** |
| --- | --- | --- | --- | --- |
| milk | HOLDK-HOLFR | 0.05 (0.04-0.07) | 0.04 (0.03-0.05) | 0.02 (0.01-0.03) |
|  | HOLDK-JER | 0.12 (0.09-0.16) | 0.15 (0.14-0.16) | 0.12 (0.07-0.15) |
|  | HOLDK-RDC | 0.12 (0.09-0.15) | 0.18 (0.16-0.19) | 0.11 (0.03-0.18) |
|  | HOLFR-JER | 0.10 (0.08-0.14) | 0.15 (0.14-0.16) | 0.12 (0.08-0.16) |
|  | HOLFR-RDC | 0.10 (0.09-0.13) | 0.16 (0.15-0.17) | 0.11 (0.03-0.18) |
|  | JER-RDC | 0.09 (0.07-0.11) | 0.20 (0.15-0.25) | 0.14 (0.10-0.15) |
| fat | HOLDK-HOLFR | 0.05 (0.04-0.07) | 0.04 (0.03-0.06) | 0.02 (0.01-0.04) |
|  | HOLDK-JER | 0.12 (0.10-0.15) | 0.20 (0.17-0.23) | 0.19 (0.13-0.26) |
|  | HOLDK-RDC | 0.12 (0.10-0.17) | 0.12 (0.10-0.14) | 0.14 (0.07-0.20) |
|  | HOLFR-JER | 0.12 (0.10-0.14) | 0.19 (0.16-0.23) | 0.18 (0.13-0.26) |
|  | HOLFR-RDC | 0.12 (0.10-0.16) | 0.12 (0.11-0.13) | 0.13 (0.08-0.18) |
|  | JER-RDC | 0.08 (0.07-0.10) | 0.27 (0.18-0.33) | 0.19 (0.12-0.28) |
| prot | HOLDK-HOLFR | 0.04 (0.03-0.06) | 0.04 (0.02-0.05) | 0.02 (0.01-0.05) |
|  | HOLDK-JER | 0.12 (0.08-0.16) | 0.18 (0.15-0.21) | 0.19 (0.13-0.30) |
|  | HOLDK-RDC | 0.11 (0.09-0.15) | 0.21 (0.20-0.21) | 0.23 (0.16-0.29) |
|  | HOLFR-JER | 0.11 (0.08-0.14) | 0.17 (0.14-0.21) | 0.18 (0.12-0.29) |
|  | HOLFR-RDC | 0.11 (0.09-0.13) | 0.20 (0.20-0.21) | 0.22 (0.16-0.28) |
|  | JER-RDC | 0.09 (0.07-0.11) | 0.18 (0.15-0.21) | 0.20 (0.14-0.27) |
